# Does probabilistic modelling of linkage disequilibrium evolution improve the accuracy of QTL location in animal pedigree?

**DOI:** 10.1186/1297-9686-42-38

**Published:** 2010-10-22

**Authors:** Christine Cierco-Ayrolles, Sébastien Dejean, Andrés Legarra, Hélène Gilbert, Tom Druet, Florence Ytournel, Delphine Estivals, Naïma Oumouhou, Brigitte Mangin

**Affiliations:** 1 INRA, UR 875 Unité de Biométrie et Intelligence Artificielle, F-31320 Castanet-Tolosan, France; 2 Université Toulouse III, UMR 5219, F-31400 Toulouse, France; 3 INRA, UR 631 Station d'Amélioration Génétique des Animaux, F-31320 Castanet-Tolosan, France; 4 INRA, UMR1313 Génétique Animale et Biologie Intégrative, F-78350 Jouy-en-Josas, France; 5 University of Liège (B43), Unit of Animal Genomics, Faculty of Veterinary Medicine and Centre for Biomedical Integrative Genoproteomics, Liège, Belgium; 6 University of Göttingen, Faculty of Agricultural Sciences, Department of Animal Sciences, Georg-August University, Göttingen, Germany

## Abstract

**Background:**

Since 2001, the use of more and more dense maps has made researchers aware that combining linkage and linkage disequilibrium enhances the feasibility of fine-mapping genes of interest. So, various method types have been derived to include concepts of population genetics in the analyses. One major drawback of many of these methods is their computational cost, which is very significant when many markers are considered. Recent advances in technology, such as SNP genotyping, have made it possible to deal with huge amount of data. Thus the challenge that remains is to find accurate and efficient methods that are not too time consuming. The study reported here specifically focuses on the half-sib family animal design. Our objective was to determine whether modelling of linkage disequilibrium evolution improved the mapping accuracy of a quantitative trait locus of agricultural interest in these populations. We compared two methods of fine-mapping. The first one was an association analysis. In this method, we did not model linkage disequilibrium evolution. Therefore, the modelling of the evolution of linkage disequilibrium was a deterministic process; it was complete at time 0 and remained complete during the following generations. In the second method, the modelling of the evolution of population allele frequencies was derived from a Wright-Fisher model. We simulated a wide range of scenarios adapted to animal populations and compared these two methods for each scenario.

**Results:**

Our results indicated that the improvement produced by probabilistic modelling of linkage disequilibrium evolution was not significant. Both methods led to similar results concerning the location accuracy of quantitative trait loci which appeared to be mainly improved by using four flanking markers instead of two.

**Conclusions:**

Therefore, in animal half-sib designs, modelling linkage disequilibrium evolution using a Wright-Fisher model does not significantly improve the accuracy of the QTL location when compared to a simpler method assuming complete and constant linkage between the QTL and the marker alleles. Finally, given the high marker density available nowadays, the simpler method should be preferred as it gives accurate results in a reasonable computing time.

## Background

For several decades, detection and mapping of loci affecting quantitative traits of agricultural interest (Quantitative Trait Loci or QTL) using genetic markers have been based only on pedigree or family information, especially in plant and animal populations where the structure of these experimental designs can be easily controlled. However, the accuracy of gene locations using these methods was limited, due to the small number of meioses occurring in a few generations. Recent advances in technology, such as SNP genotyping, leading to dense genetic maps have boosted research in QTL detection and fine-mapping. Nowadays, methods for fine-mapping rely on linkage disequilibrium (LD) information rather than simply on linkage data. Linkage disequilibrium, the non-uniform association of alleles at two loci, has been successfully employed for mapping both Mendelian disease genes [1-4] and QTL [5-7]. Interested readers can also refer to reviews by [8-11]. For all chromosomal loci, including those that are physically unlinked, linkage disequilibrium can be generated or influenced by various evolutionary forces such as mutation, natural or artificial selection, genetic drift, population admixture, changes in population size (exponential growth or bottleneck, for instance). Most methods using the linkage disequilibrium concept for QTL fine-mapping are based on the genetic history of the population. Whichever method is used to include population genetics concepts (calculation of Identity By Descent (IBD) probabilities under given assumptions about population history [6], Wright-Fisher based allele frequency model [12], backward inferences through the coalescent tree [13]), computation is always time consuming. Furthermore, since mapping accuracy depends on the length of the haplotype used in the study [14-17], this computational time could become prohibitive when many markers are being considered. Therefore, with new technologies such as SNP genotyping and the amount of data they generate, it is interesting to evaluate the improvement in accuracy produced by these time consuming methods opposed to using simpler methods. In this study, we focused on animal populations of agricultural interest. Generally, these populations have a small effective size, and are composed of a few families with about a hundred descendants.

We considered that a dense genetic map was available. Our main objective was to compare the QTL prediction accuracy of two methods in the half-sib family design. These two methods differed in the way they modelled the evolution of linkage disequilibrium between a QTL and its flanking markers, through the probability of bearing the favourable QTL allele given the marker observations. The first method, HaploMax, was a haplotype-based association analysis, very similar to the one developed by Blott et al. [7]. In this method, there was no specific modelling of linkage disequilibrium evolution: linkage disequilibrium was complete at time 0 on the mutated haplotype and remained complete during the following generations. Therefore, the probability of bearing the favourable QTL allele given the mutated haplotype is always equal to one during the generations. This is why we mentioned the deterministic evolution of linkage disequilibrium. The second method, HAPimLDL, was a maximum likelihood approach [12] and it used probabilistic modelling of the temporal evolution of linkage disequilibrium based on a Wright-Fisher model. This probabilistic modelling of the temporal evolution of linkage disequilibrium made it possible to vary the probability of bearing the favourable QTL allele given the marker informations during generations. Our hypothesis was that, in these animal populations with a small effective size and having evolved over a few generations, a rough model based on the deterministic evolution of linkage disequilibrium was as accurate as a probabilistic-based model and should therefore be preferred from a computational point of view. Both methods assumed a single QTL effect for all the families. Both allow any number of flanking markers to be considered using a sliding window across a previously identified QTL region. Both methods have been implemented in an R-package freely available from the Comprehensive R Archive Network (CRAN, http://cran.r-project.org/).

In this paper, we have considered only half-sib family designs. In this framework, we used simulations to compare the performance of these two fine-mapping methods. We investigated the effect of various scenarios on the performance of the methods: allelic effect of the QTL, marker density, population size, mutation age, family structure, selection rate, mutation rate and number and size of the families. For each of these scenarios, we investigated the improvement produced by probabilistic modelling of linkage disequilibrium evolution.

## Methods

The genetic model used in this paper was described by [18]. The population was considered as a set of independent sire families, all dams being unrelated to each other and to the sires. We considered a bi-allelic QTL with additive effect only and a single QTL effect for all the families. We assumed the same phase across families. We will only briefly describe the HaploMax method, as it is a standard method. The HAPimLDL method, which has been developed for this work, is presented in detail.

### The HaploMax method

HaploMax is a marker-haplotype-regression method adapted to the following two hypotheses: the QTL is bi-allelic, and QTL alleles and marker alleles are in complete linkage. In each marker interval, and for each flanking marker haplotype, we performed a haplotype-based association analysis with a sire effect and a dose haplotype effect (0 for absence of the haplotype, 1 for one copy of the haplotype, 2 for homozygosity). We tested each haplotype in turn against all the others [7] and the HaploMax value was given by the haplotype maximising the F-test values.

The HaploMax method is therefore perfectly suited to demonstrate the effect of a causal bi-allelic mutation. In HaploMax, there was no probabilistic modelling of linkage disequilibrium evolution. Linkage disequilibrium was complete at time 0 and remained complete during the following generations.

### The HAPimLDL method for half-sib family designs

This likelihood-based method is detailed in the following sub-sections. It combines family information with probabilistic modelling of linkage disequilibrium evolution (LDL stands for Linkage and Linkage Disequilibrium). For clarity purposes, some of the longer calculations are presented in the Appendix.

#### Notation

A bi-allelic QTL is assumed with alleles *Q *and *q*.

Let *i *(*i *= 1, ..., *I *) be the identification of a family. Let *ij *(*j *= 1, ..., *n*_*i*_) be the index of a mate of sire *i *(*i *= 1, ..., *I *) and *ijk *(*k *= 1, ..., *n*_*ij *_) denote the progeny of dam *ij*. When considering strictly half-sib families, only one progeny is measured per dam (*n*_*ij *_= 1) (in the case of bovine populations, for instance), and the *k *index can be omitted.

Assuming that the available information consists of the phenotypic value of each progeny and a set of haplotypes of observed markers aligned on a genetic map, we can establish the following notations:

• $h_{i}=(h_{i}^{1},h_{i}^{2})$, marker haplotypes of sire *i*. $h_{i}^{1}$ (respectively $h_{i}^{2}$ ) is the set of marker alleles carried by the first (respectively second) chromosome of the sire *i*,

• $h_{ij}=(h_{ij}^{s},h_{ij}^{d})$, marker haplotypes of progeny *ij *transmitted respectively by its father and mother,

• *y_ij _*, phenotype of progeny *ij*.

If *x *denotes a putative bi-allelic QTL locus on the genome:

• $Z_{i}(x)=Q_{i}^{1}(x)Q_{i}^{2}(x)$, the sire diplotype at locus *x*, where $Q_{i}^{1}(x)$ and $Q_{i}^{2}(x)$ denote the QTL allele at locus *x *carried respectively by the two homologous chromosomes. Note that there are three genotypes but four diplotypes since there are two heterozygous diplotypes (*Qq *and *qQ*).

• $\tilde{h_{i}(x)}=\tilde{\left(h_{i}^{1}(x\right)},\tilde{h_{i}^{2}(x)})$, marker and locus *x *haplotypes of sire *i*. This is the extended marker haplotype of sire *i *including the alleles at the QTL locus *x *.

• $Q_{ij}^{d}(x)$, the allele at the QTL locus *x *transmitted by the dam *ij *to her single progeny,

• $Q_{ij}^{s}(x)$, the allele at the QTL locus *x *transmitted by the sire *i *to his progeny *ij*.

#### LDL likelihood

The population was considered as a set of independent sire families, all dams being unrelated both to each other and to the sires. The likelihood is constructed as follows: a Gaussian mixture models the phenotypes as a function of QTL states. These are unknown, but their probability depends on the surrounding markers through LD, which is modelled by the Wright-Fisher model. Further, if the chromosome has been received from a sire, the probability of descent of each paternal chromosome is considered. Let Λ*_ij_*(*x*) denote the individual *ij *likelihood.

$$\begin{array}{l} \Lambda_{ij}(x)=\text{d}\mathbb{P}(Y_{ij}=y_{ij}|h_{ij},h_{i})(x)\\ =\sum_{z=1}^{4}\mathbb{P}(Z_{i}(x)=z|h_{ij},h_{i})\\ \;\;\times \sum_{a=1}^{2}\left[\mathbb{P}(Q_{ij}^{d}(x)=a|h_{ij},h_{i})\text{d}\mathbb{P}(Y_{ij}=y_{ij}|h_{ij},h_{i},Z_{i}(x)=z,Q_{ij}^{d}(x)=a)\right]\\ =\sum_{z=1}^{4}\mathbb{P}(Z_{i}(x)=z|h_{i})\sum_{a=1}^{2}\mathbb{P}(Q_{ij}^{d}(x)=a|h_{ij}^{d})\\ \times \left(\begin{array}{ll} \varphi (y_{ij};\mu_{i}+\alpha_{Qa},\sigma^{2}) & \left(\mathbb{P}(Q_{ij}^{s}(x)\leftarrow Q_{i}^{1}(x)|\tilde{h_{i}(x)},h_{ij}^{s})\mathbb{P}(Q_{i}^{1}(x)=Q|Z_{i}(x)=z\right)\\ & +\mathbb{P}(Q_{ij}^{s}(x)\leftarrow Q_{i}^{2}(x)|\tilde{h_{i}(x)}),h_{ij}^{s})\mathbb{P}(Q_{i}^{2}(x)=Q|Z_{i}(x)=z))\\ +\varphi (y_{ij};\mu_{i}+\alpha_{qa},\sigma^{2}) & \left(\mathbb{P}Q_{ij}^{s}(x)\leftarrow Q_{i}^{1}(x)|\tilde{h_{i}(x)},h_{ij}^{s})\mathbb{P}(Q_{i}^{1}(x)=q|Z_{i}(x)=z\right)\\ & +\mathbb{P}(Q_{ij}^{s}(x)\leftarrow Q_{i}^{2}(x)|\tilde{h_{i}(x)},h_{ij}^{s})\mathbb{P}(Q_{i}^{2}(x)=q|Z_{i}(x)=z)) \end{array}\right) \end{array}$$

where

• *z *= 1, 2, 3 and 4 stands for *QQ, qq, Qq *and *qQ *respectively,

• *a *= 1 and 2 for *Q *and *q*,

• *μ_i _*is the *phenotype *mean within the sire family *i*, and *σ*^2 ^the residual variance,

• *φ*(·; *μ*, *σ*^2 ^) is *the *Gaussian probability density function with mean *μ *and variance *σ*^2^

• for *a *= 1 and 2, the *α_Qa _*and *α_qa _*parameters, subject to the constraint of their sum being equal to 0, are the effects of the diplotypes at locus *x*. The constraint *α_qQ _*= *α_Qq _*= 0 leads to an additive model

• the symbol " ←" in the quantities $\mathbb{P}(Q_{ij}^{s}(x)\leftarrow Q_{i}^{k}(x)|\tilde{h_{i}(x)},h_{ij}^{s})$ means "comes from".

In this likelihood, the probabilities due to linkage that are contained in the transmission probabilities $\mathbb{P}(Q_{ij}^{s}(x)\leftarrow Q_{i}^{k}(x)|\tilde{h_{i}(x)},h_{ij}^{s})$ for *k *= 1, 2 were computed using QTLMAP subroutines that implement the approximate method described in [18].

The expression above considers QTL effects, probabilities of transmission of QTL alleles from sires to offspring, and probabilities of QTL states in the founders. The linkage disequilibrium signal comes from the quantities ℙ(*Z_i _*(*x*) = *z*|*h_i_*) and $\mathbb{P}(Q_{ij}^{d}(x)=a|h_{ij}^{d})$ which are the probabilities of QTL alleles in the parents conditional on the surrounding marker haplotypes. QTL diplotype probabilities given marker information, contained in ℙ(*Z_i_*(*x*) = *z*|*h_i_*), were computed assuming the Hardy-Weinberg equilibrium. Thus,

$$\begin{array}{l} \mathbb{P}(Z_{i}(x)=QQ|h_{i})=\mathbb{P}(Q_{i}^{1}(x)=Q|h_{i}^{1})\mathbb{P}(Q_{i}^{2}(x)=Q|h_{i}^{2})\\ \mathbb{P}(Z_{i}(x)=Qq|h_{i})=\mathbb{P}(Q_{i}^{1}(x)=Q|h_{i}^{1})\mathbb{P}(Q_{i}^{2}(x)=q|h_{i}^{2})\\ \mathbb{P}(Z_{i}(x)=qQ|h_{i})=\mathbb{P}(Q_{i}^{1}(x)=q|h_{i}^{1})\mathbb{P}(Q_{i}^{2}(x)=Q|h_{i}^{2})\\ \mathbb{P}(Z_{i}(x)=qq|h_{i})=\mathbb{P}(Q_{i}^{1}(x)=q|h_{i}^{1})\mathbb{P}(Q_{i}^{2}(x)=q|h_{i}^{2}) \end{array}$$

QTL allelic probabilities given marker information for both sire and dam were computed under the linkage disequilibrium model described in the next section.

The probability terms, $\mathbb{P}(Q_{i}^{j}(x)=Q|Z_{i}(x)=z)$ and $\mathbb{P}(Q_{i}^{j}(x)=q|Z_{i}(x)=z)$ (*j *= 1, 2), involving sire QTL allele given sire QTL diplotype, are either 0 or 1.

#### Likelihood approximation and linkage disequilibrium model

QTL allelic probabilities given marker information for the parents are terms that are modelled through the evolution of linkage disequilibrium across generations. These terms depend on the frequencies of marker haplotypes and on the frequencies of QTL allele and marker extended haplotypes. Under traditional models of population genetics, these haplotype frequencies are stochastic. Thus, the likelihood function cannot be easily calculated and must be approximated. Following [12], we used the likelihood given the expected value of haplotype frequencies to approximate the overall expected value of the likelihood and we limited marker haplotypes to a small number of markers surrounding the putative QTL locus (in our study, we considered either two flanking markers or four flanking markers). This led to the following approximations for *a *= 1, 2 and *k *= 1, 2:

$$\begin{array}{c} \mathbb{P}(Q_{i}^{k}(x)=a|h_{i}^{k})\approx \min\left(1,\frac{E[\Pi_{a,hIM_{i}^{k}}(t)]}{E[\Pi_{hIM_{i}^{k}}(t)]}\right)\\ \mathbb{P}(Q_{ij}^{d}(x)=a|h_{ij}^{d})\approx \min\left(1,\frac{E[\Pi_{a,hIM_{ij}^{d}}(t+1)]}{E[\Pi_{hIM_{ij}^{d}}(t+1)]}\right) \end{array}$$

where

• $hIM_{i}(t)=(hIM_{i}^{1}(t),hIM_{i}^{2}(t))$ denotes the haplotypic pair limited to markers surrounding the locus *x *carried by sire *i *at time *t *and, $\Pi_{hIM_{i}^{k}}(t)$ the frequency of the haplotype mentioned.

• $\Pi_{a,hIM_{i}^{k}}(t)$ is the frequency of sire *i *haplotypes carrying both the *a *allele at the *x *locus and the haplotype $hIM_{i}^{k}$ at the flanking markers at time *t*.

• $hIM_{ij}^{d}(t+1)$ denotes the progeny *ij *haplotype at time *t *+ 1 transmitted by its mother and limited to markers surrounding the *x *locus. $\Pi_{hIM_{ij}^{d}}(t+1)$ is the corresponding frequency,

• $\Pi_{a,hIM_{ij}^{d}}(t+1)$ is the frequency of progeny *ij *haplotypes carrying both the *a *allele at the *x *locus and the haplotype $hIM_{ij}^{d}(t+1)$ at the flanking markers at time *t *+ 1.

These haplotype frequencies at time *t *could be expressed as functions of marker frequencies, digenic, trigenic... disequilibria at time *t *[19]. Moreover, under the hypotheses of a Wright-Fisher model, no interference and a large population size, the expected values of marker frequencies and disequilibria at time *t *could be derived from the same quantities at time 0 and the recombination rates between the QTL locus and the markers [19,20]. Therefore, we generalised the formula obtained by [12] in order to take into account any number of surrounding markers. These calculations are detailed in the Appendix.

Finally, we had to model the haplotype frequencies at time 0. Following [12], we assumed an initial creation of linkage disequilibrium that was due to mutation or migration. Generally speaking, assuming that the *Q *allele at time 0 appeared on a haplotype denoted h*, then the time zero model was

$$\Pi_{h,Q}(0)=(1-\beta )\Pi_{h}\Pi_{Q}(0)+\beta \Pi_{Q}(0)\delta_{h=h^{*}}$$

where the parameter *β *represents the proportion of new copies of allele *Q *introduced at time 0, *δ_x = y _*is the Kronecker delta operator (equal to 1 if *x *= *y *and 0 otherwise), Π*_h,Q_*(0) and Π*_Q_*(0) are the frequencies of the haplotypes (*h*, *Q*) and *h *at time 0, and Π*_h _*is the frequency of haplotype *h*.

In our specific study, we simplified the time 0 model assuming that there was no pre-existing copy of the *Q *allele and we set *β *equal to 1.

### HAPim R-package

From a computational point of view, the HAPimLDL likelihood calculation was divided into two parts. In the first part, devoted to the calculation of transmission probabilities and the reconstruction of sire and progeny chromosomes, we used a modified version of the software QTLMAP written in Fortran 95 [18]. The second part aimed at calculating and maximizing the likelihood in the half-sib design. It was developed using the R free software environment for statistical computing [21]. An R package named "HAPim" was implemented and is freely available from the Comprehensive R Archive Network (CRAN, http://cran.r-project.org/).

### Simulations

Simulations were carried out in order to compare these methods in the specific design of half-sib families. For each simulation, 500 replicates were performed.

The populations were simulated using the LDSO (Linkage Disequilibrium with Several Options) program developed in Fortran 90 by [22] and based on the gene-dropping method [23]. There was no constraint on the QTL frequency, but we discarded simulations for which there was no heterozygous sire. Evolution of the founder population was modelled through two parameters: the effective size (i.e. the number of founders) and the time of evolution. We studied two extreme scenarios for the founder population. In the first, at time 0, we assumed complete linkage disequilibrium of QTL-markers (by introducing a mutation in a single haplotype) and linkage equilibrium between markers. In the second scenario, the QTL and the markers were at equilibrium. Evolution time was 50 generations in almost all simulations, except a 200 generation evolution time in one case of the "disequilibrium scenario" and a 100 generation evolution time in one case of the "equilibrium scenario". We considered three effective population size values: 100, 200 and 400. In most simulations we did not assume selection, mutation, or bottleneck. However, to investigate the robustness of the methods, three simulations were also performed to study the effect of selection and one to study the influence of mutation.

We simulated a set of half-sib families. Two parameters- the number of sires (equal to 10, 20, 25, 50 or 100) and the number of progeny per sire (equal to 10, 20, 25,50 or 100)- were varied to address the problem of how to choose between many small families and a few large families.

All simulations were compared both to each other and to the reference simulation. In the reference simulation, we considered a 10 cM chromosomal area with 40 evenly spaced bi-allelic markers and a population size of 100 evolving over 50 generations. We simulated a set of 20 sires, each having 100 progeny. A single QTL with a substitution effect of 0.25 was simulated at a position of 3.35 cM. We then varied the different parameters with respect to this reference simulation in order to assess their respective influence. We considered three different values of map density (0.125 cM, 0.25 cM and 0.5 cM). The phenotypic values were simulated with a fixed dose-response model at the QTL position (i.e. regression model as a function of the number of Q alleles) and a residual variance of 1.

In the first set of simulations, presented in Tables 1 and 2, we analyzed only three-locus haplotypes (composed of the QTL and its two flanking markers). In Table 3, we also conducted simulations where the haplotype length was equal to 5 (the QTL and two flanking markers on both sides of the QTL).

## Results

In the following tables, we present square roots of the mean square error (MSE) of the QTL position. The MSE value is given by the following formula

$$MSE(s)=\frac{\sum_{r=1}^{500}{\left({\hat{s}}_{r}-s\right)}^{2}}{500}$$

where ${\hat{s}}_{r}$ is the estimated QTL position in replicate *r*, *s *is the true QTL position and 500 is the total number of replicates. We also computed the mean absolute error criterion and found a clear linear dependency between these two criteria (data not shown).

We compared the two methods, HaploMax and HAPimLDL, with a t-test on the MSE values and found no significant difference between them for any of the scenarios studied.

### Complete linkage disequibrium between the QTL and the markers

In this set of simulations we simulated the scenario for which there were complete linkage disequilibrium QTL-markers and linkage equilibrium between markers in the founder population.

#### Influence of genetic and population parameters

Here we describe the sensitivity of the two methods to the following parameters: QTL allelic effect value, marker density, population's effective size of population, number of generations, mutation and selection. However, despite the fact that our goal was the accuracy of location, we computed some power values for both methods, the 5% thresholds being obtained by permutation. For the reference simulation, the power value was equal to 63% for Haplomax and to 56% for HAPimLDL. The highest power values were obtained for the QTL value equal to 0.5 and were around 90% for both methods. The lowest power values were obtained when *N_e _*was equal to 400 and *N_g _*equal to 50, and were around 15%. Table 1 summarises the simulation results. It is not surprising to see that the bigger the QTL allelic effect, the more accurate the method. The marker density had only a very slight influence on the MSE value. HaploMax presented an erratic trend with the marker density. HAPimLDL showed a clear decrease in the MSE values with increasing marker density.

With regard to the design parameters, we noticed that the precision of the QTL position decreased as the sample size (i.e. number of sires × number of progeny per sire) decreased, regardless of the family structure. For a fixed number of generations, the MSE values increased as the effective size of the population increased. However, when both effective size and number of generations varied, provided that their ratio remained constant, MSE values were not modified, which is completely consistent with traditional theory in population genetics.

When we allowed all SNP markers to mutate at a mutation rate equal to 10^-6^, we found a loss of accuracy of about 20-25% for HaploMax and about 50% for HAPimLDL (data not shown). In this case, the power value was equal to 59% for HaploMax and to 49% for HAPimLDL.

**Table 1:** Square roots of MSE values (in cM) for both methods, HaploMax and HAPimLDL, under various scenarios

Method	Param	Ref simul	QTL effect	Marker density		Sample size		Effective size		
	QTL	0.25	0.5							
	*N_e_*	100						200	400	400
	*N_g_*	50								200
	*N_s_*	20				20	10			
	*N_p_*	100				50	50			
	*dens*	0.25		0.125	0.5					

HaploMax		2.018	1.431	2.138	2.134	2.496	2.774	2.493	2.840	2.054
HAPimLDL		2.165	1.528	2.114	2.296	2.716	2.990	2.635	2.834	2.147

Square roots of MSE values (in cM) for both methods, HaploMax and HAPimLDL, under various scenarios; we assumed complete linkage disequilibrium between the QTL and the markers and linkage equilibrium between the markers in the founder population; the haplotype is composed of the QTL and two flanking markers; the true QTL position is 3.35 cM on a 10 cM-long chromosomal region; unspecified parameters are equal to the corresponding parameters in the reference simulation; in this table, QTL denotes the QTL allelic effect value, *N_e _*is the effective size of the population, *N_g _*is the number of generations, *N_s _*is the number of sires, *N_p _*is the number of progeny per sire and *dens *is the marker density; each scenario was simulated 500 times

#### Influence of phenotypic selection

The influence of phenotypic selection is presented in Table 2. We considered two values for the additive QTL effect and two selection strengths (light and strong).

The QTL effect had no influence on the accuracy of location. However, selection led to a loss of accuracy of about 50% with light selection and 60% with strong selection. On the one hand, the selection causes a hitchhiking effect which amplifies the signal from the region where the QTL is located but, on the other hand, it widens this region, leading to a loss of accuracy (higher MSE values). For example, a possible outcome of selection is that just a few different haplotypes are carriers of the *Q *allele. This loss of accuracy had already been pointed out by [24]. It was concluded that selection increased MSE values, leading to large confidence intervals of the QTL position, and therefore to additional difficulties in locating the mutation. Moreover, the power values collapsed in this situation (around 4% for both methods with strong selection and around 13% for both methods with light selection).

**Table 2:** Square roots of MSE values (in cM) for both methods in the presence of phenotypic selection

Method	Param	Ref Simul	Strong selection	Light selection	Light selection
	QTL	0.25			0.5
	*N_e_*	100			
	*N_g_*	50			
	*N_s_*	20			
	*N_p_*	100			
	*dens*	0.25			
	*sel*	No selection	*sel *= 0.5	*sel *= 0.8	*sel *= 0.8

HaploMax		2.018	3.403	3.125	3.103

HAPimLDL		2.165	3.306	3.151	3.124

Square roots of MSE values (in cM) for both methods in the presence of phenotypic selection; we assumed complete linkage disequilibrium between the QTL and the markers and linkage equilibrium between the markers in the founder population. The haplotype is composed of the QTL and two flanking markers; the true QTL position is 3.35 cM on a 10-cM long chromosomal region; unspecified parameters are equal to the corresponding parameters in the reference simulation; in this table, QTL denotes the QTL allelic effect value, *N_e _*is the effective size of the population, *N_g _*is the number of generations, *N_s _*is the number of sires, *N_p _*is the number of progeny per sire, *dens *is the marker density and *sel *denotes the selection parameter; each scenario was simulated 500 times

#### Influence of haplotype length and population structure

In Table 3, we studied the influence of haplotype length on the accuracy of the QTL location. It is clear that there is a significant gain when using four markers instead of two. All the previous conclusions remained valid when using four markers. If four markers were used in the model, increasing the sample size seemed to be the only way to decrease the MSE.

The influence of the population structure itself is also investigated in Table 3. Since we noted that haplotypes containing four markers led to the best results, we have focused the discussion only on this type of haplotype. Through this set of simulations, we have tried to resolve the issue of whether it is better to study many small families or a few large families. The results are in favour of having many founders, which increases the power value. However, this is only clear when both the sample size and the number of markers are large.

**Table 3:** Square roots of MSE values (in cM) for both methods for two haplotype lengths: the QTL and its two flanking markers and the QTL and its four flanking markers

Param		Methods			
		**HaploMax**		**HAPimLDL**	

**Number of markers**		**2**	**4**	**2**	**4**

*N_s_*	20	1.66	1.26	1.66	1.26
*N_p_*	100				

*N_s_*	100	1.65	1.11	1.71	1.15
*N_p_*	20				

*N_s_*	20	1.68	1.36	1.74	1.45
*N_p_*	50				

*N_s_*	50	1.73	1.32	1.83	1.46
*N_p_*	20				

*N_s_*	20	1.73	1.39	1.81	1.47
*N_p_*	25				

*N_s_*	25	1.83	1.49	1.85	1.59
*N_p_*	20				

*N_s_*	50	1.82	1.57	1.98	1.53
*N_p_*	10				

*N_s_*	10	1.85	1.41	1.92	1.61
*N_p_*	50				

Square roots of MSE values (in cM) for both methods for two haplotype lengths: the QTL and its two flanking markers and the QTL and its four flanking markers; we assumed complete linkage disequilibrium between the QTL and the markers and linkage equilibrium between the markers in the founder population; the true QTL position is 3.35 cM on a 10-cM long chromosomal region; the QTL allelic effect value is equal to 1, the effective size of the population is equal to 100, the number of generations is equal to 50 and the marker density is equal to 0.5 cM; *N_s _*is the number of sires and *N_p _*is the number of progeny per sire; each scenario was simulated 500 times

### The equilibrium case

In this section, we simulated a scenario where the QTL and the markers were at equilibrium in the founder population. We only varied the effective size (50 or 100) and the number of generations (50 or 100) with respect to the reference simulation. Results are presented in Table 4. We noted that MSE values in Table 4 are lower than the corresponding MSE values in Table 1. This was not surprising since, in the situation where the QTL and the markers were at equilibrium, there were more sires carrying the favourable QTL allele than in the "complete disequilibrium" case studied in Table 1. Moreover, the HaploMax method again gave MSE values slightly below those given by the HAPimLDL method. Finally, we noticed that MSE increased when the effective size decreased or the number of generations increased. This is also completely coherent since, in this situation, allelic frequencies have moved towards fixation.

**Table 4:** Square roots of MSE values (in cM) for both methods

Method	Param	Ref simul	Number of generations	Effective size
	QTL	0.25		
	*N_e_*	100		50
	*N_g_*	50	100	
	*N_s_*	20		
	*N_p_*	100		
	*dens*	0.25		

HaploMax		1.49	1.85	1.69
HAPimLDL		1.65	1.98	1.85

Square roots of MSE values (in cM) for both methods in the case where the QTL and the markers were at equilibrium in the founder population; the haplotype is composed of the QTL and two flanking markers; the true QTL position is 3.35 cM on a 10-cM long chromosomal region; unspecified parameters are equal to the corresponding parameters in the reference simulation; in this table, QTL denotes the QTL allelic effect value, *N_e _*is the effective size of the population, *N_g _*is the number of generations, *N_s _*is the number of sires, *N_p _*is the number of progeny per sire, *dens *is the marker density; each scenario was simulated 500 times

## Discussion

Within a dense genetic map framework, we have compared two QTL mapping methods aiming at locating one QTL on a chromosome in half-sib family designs. On the one hand, in the HaploMax method there was no specific modelling of linkage disequilibrium evolution and the probability of bearing the favourable QTL allele given the mutated haplotype was always equal to one during the generations. On the other hand, in the HAPimLDL method we used a probabilistic modelling of the temporal evolution of linkage disequilibrium. In this latter method, the probabilistic modelling allowed a temporal evolution of the conditional probability of bearing the favourable QTL allele given the marker observations. Our simulated scenarios mimicked animal populations shortly after creation of the breed (i.e. small populations with a short evolution time). We compared our results with those of [25], leading to conclusions very similar to theirs: very slight influence of marker density on the mapping accuracy, mapping accuracy increasing with sample size, QTL effect, number of generations since mutation occurrence, and effective size. However, although we achieved results of the same order of magnitude, slight differences in MSE values were observed mainly due to the following three reasons: we did not study exactly the same type of population; [25] assumed that haplotypes were known, but we reconstructed them; and, finally, we did not consider the same value for the number of generations parameter. It has been established that the evolution time parameter has a great influence on the accuracy of the location [[25], table five]. Despite these differences, and despite the fact that one of our methods took into account the transmission from sires to sibs, both studies showed the same tendencies with regard to the mapping accuracy. We found a gain in mapping accuracy when using a 4-SNP haplotype instead of a 2-SNP one. However, this result is valid with a fixed density marker (the one we used in our simulation study). With a very high density marker, a 1-SNP haplotype will probably lead to the best results. Finally, we demonstrated that neither method was robust to selection. The simulations showed that both methods led to similar results concerning QTL position accuracy. The simplest method, HaploMax, performed as well as HAPimLDL. This is in agreement with recent findings. In [26], it has also been concluded that a three-marker-haplotype-based association analysis (deterministic complete LD modelling) could be as efficient as the IBD method of [6]. The conclusion of our study is that the probabilistic modelling of the linkage disequilibrium evolution using a Wright-Fisher model did not improve the accuracy of the QTL location when compared to a simple method using deterministic modelling that assumed complete and constant linkage between the QTL and the marker alleles. The deterministic model, which is a rough model, was efficient enough in our simulated scenarios, which mimicked animal populations shortly after the creation of the breed (i.e. small populations with a short evolution time).

The conclusion might then be to use HaploMax for animal populations with a small effective size and having evolved over a few generations. In fact, the forward method associated with causal mutation, used in our simulation study, reflected exactly the theoretical evolution model used to compute the LD dynamics in the likelihood function, thus favouring the HAPimLDL method as against the HaploMax method. Therefore, we can conclude that the HAPimLDL method did not perform significantly better than simpler methods within our evolution scenarios.

When dealing with populations with large effective sizes or with very old mutations, combining linkage with probabilistic modelling of linkage disequilibrium evolution should produce the greatest accuracy. Actually, in these populations, a huge number of recombination events would occur, leading to a small extent of the linkage disequilibrium signal. Therefore, deterministic complete linkage disequilibrium modelling would be less appropriate in this case.

## Competing interests

The authors declare that they have no competing interests.

## Authors' contributions

BM coordinated the whole LDLmapQTL project. CCA, AL and BM developed the methods, designed the simulation study, analyzed the simulation results and wrote the paper. FY, HG and TD were responsible for the LDSO program. DE implemented the HAPimLDL method. NO performed the simulation study. SD,NO, DE and BM created the R package. All authors read and approved the final manuscript.

## Appendix

To derive haplotype frequencies at time *t *as functions of haplotype frequencies at time 0, we used the Bennett decomposition of haplotype frequencies [19] and the work of [20].

Let *A_n _*denote a set of *n *alleles at *n *different loci, *A_n _*= {*a*_1_, *a*_2_, ..., *a_n_*}. Let *D_n_*(*A_n _*, *t*) be the n-loci linkage disequilibrium of *A_n _*alleles at time *t *defined by [19] such that, in an infinitely large population, under random mating and meiosis

(1) $$D_{n}\left(A_{n},t+\text{1}\right)=\rho_{\{A_{n}}{}_{\}}D_{n}\left(A_{n},t\right)$$

where *ρ*{*A_n _*} is the probability of no recombination across loci belonging to *A_n _*.

Assuming no interference between loci leads to

$$\rho_{\left\{A_{n}\right\}}=\prod_{i=1}^{n-1}\left(1-c_{i,i+1}\right)$$

where *c_i, i' _*is the recombination rate between loci *i *and *i'*.

Let $\Pi_{A_{n}}$ (*t*) be the frequency of the haplotype carrying the alleles in *A_n _*at time *t*. Then by definition

(2) $$\Pi_{A_{n}}(t)=\sum_{p=\left\{\cup_{i}A_{n_{i}}=A_{n}\right\}}\bar{C_{p}}\left(\prod_{i}D_{ni}(A_{ni},t)\right)$$

where the coefficients $\bar{C_{p}}$ are constants obtained by recursion [20], and *p *= {⋃*_i _A_ni _*= *A_n_*} denotes a partition of *A_n_*. For example, for *n *= 3 there are 5 partitions namely {*a*_1_, *a*_2_, *a*_3_}, {{*a*_1_, *a*_2_}⋃{*a*_3_}}, {{*a*_1_, *a*_3_}⋃{*a*_2_}}, {{*a*_2_, *a*_3_}⋃{*a*_1_}} and {{*a*_1_}⋃{*a*_2_}⋃{*a*_3_}}.

When *n *equals two and three, [20] proved that the $\bar{C_{p}}$ are all equal to one. But when *n *≥ 4, some $\bar{C_{p}}$ are not equal to one even if we assume no interference between loci. For example, for the partition {{*a*_1_, *a*_4_}⋃{*a*_2_, *a*_3_}} with four loci, [20] proved that

$${\bar{c}}_{\left\{\{a_{1},a_{4}\}\cup \{a_{2},a_{3}\}\right\}}=\frac{c_{12}c_{34}}{\left(1-c_{14})-(1-c_{12})(1-c_{34}\right)}$$

which does not reduce to unity, except for unlinked loci. This means that, for *n *≥ 4, the Bennett disequilibria are different from disequilibria defined by [27-29] since these authors imposed $\bar{C_{p}}$ = 1 in formula (2). However, the Bennett disequilibria are the only multilocus linkage disequilibrium measures that decay geometrically with time.

Let *n *be odd and composed of (*n *− 1)/2 left and right markers surrounding a putative causal locus. Assume that at time 0 all the Bennett disequilibria between markers are null, i.e. markers were in equilibrium when the causal mutation appeared. Formula (1) states that marker disequilibria are null throughout the population history. Moreover, all the terms not equal to zero in the formula (2), applied to the frequency of markers and the mutated locus haplotypes, have a $\bar{C_{p}}$ constant equal to one. Partitions that do not involve marker disequilibria are such that

$$p=\left\{\underset{k}{\cup }\left\{a_{k}\right\}\cup A_{p}=A_{n}\right\}$$

where the causal locus is in the set *A_p _*and *k *= 0 means *A_p _*= *A_n_*. Since those partitions are composed of singletons and a single subset of *A_n_*, $\bar{C_{p}}$ = 1 (formula 4.14 in [20]), then we get

(3) $$\Pi_{A_{n}}(t)=\sum_{p=\{\cup_{k}\{a_{k}\}\cup A_{p}=A_{n}\}}{\left(\rho_{\left\{A_{p}\right\}}\right)}^{t}D_{\#;A_{p}}(A_{p},0)\left(\prod_{k}\Pi_{a_{k}}(0)\right)$$

where #*A*_*p *_denotes the cardinal of set *A*_*p *_. We finish the calculation by using the reverse formula of *D*_#*Ap *_(*A*_*p *_, 0) as a function of haplotype frequencies at time 0, which in this case can be obtained easily using recursion based on the following equation

(4) $$D_{n}(A_{n},0)=\Pi_{A_{n}}(0)-\sum_{\underset{k\neq 0}{p=\{\cup_{k}\{a_{k}\}\cup A_{p}=A_{n}\}}}D_{\#;A_{p}}(A_{p},0)\left(\prod_{k}\Pi_{a_{k}}(0)\right)$$

In a finite population, formulae developed in an infinite population, can be transformed using the expectation of multi-locus disequilibria and haplotype frequencies, and taking only the first order development of these expectations as the population size extends to infinity. We then get

(5) $$E[\Pi_{A_{n}}(t)]≃\sum_{p=\{\cup_{k}\{a_{k}\}\cup A_{p}=A_{n}\}}{\left(\rho_{\left\{A_{p}\right\}}\right)}^{t}D_{\#A_{p}}(A_{p},0)\left(\prod_{k}\Pi_{a_{k}}(0)\right)$$

where ≃ means asymptotically equivalent.

Equalities of first order developments are based on the fact that products of expectations are asymptotically equal to expectations of products. These equalities can also be found using the work of [27].
